# New Insights into Molecular Links Between Microbiota and Gastrointestinal Cancers: A Literature Review

**DOI:** 10.3390/ijms21093212

**Published:** 2020-05-01

**Authors:** Yash Raj Rastogi, Adesh K. Saini, Vijay Kumar Thakur, Reena V. Saini

**Affiliations:** 1 School of Bioengineering and Food Technology, Faculty of Applied Sciences and Biotechnology, Shoolini University of Biotechnology and Management Sciences, Solan, Himachal Pradesh 173229, India; iamyashraj0@gmail.com; 2 Faculty of Sciences, Shoolini University of Biotechnology and Management Sciences, Solan, Himachal Pradesh 173229, India; adeshsaini@shooliniuniversity.com; 3 Biorefining and Advanced Materials Research Centre, Scotland’s Rural College (SRUC), Kings Buildings, Edinburgh, EH9 3JG, UK; 4 School of Biotechnology, Faculty of Applied Sciences and Biotechnology, Shoolini University of Biotechnology and Management Sciences, Solan, Himachal Pradesh 173229, India

**Keywords:** microbiota, Wnt-β-catenin signalling, colorectal cancer, *Fusobacterium nucleatum*, TLRs, butyrate

## Abstract

Despite decades of exhaustive research on cancer, questions about cancer initiation, development, recurrence, and metastasis have still not been completely answered. One of the reasons is the plethora of factors acting simultaneously in a tumour microenvironment, of which not all have garnered attention. One such factor that has long remained understudied and has only recently received due attention is the host microbiota. Our sheer-sized microbiota exists in a state of symbiosis with the body and exerts significant impact on our body’s physiology, ranging from immune-system development and regulation to neurological and cognitive development. The presence of our microbiota is integral to our development, but a change in its composition (microbiota dysbiosis) can often lead to adverse effects, increasing the propensity of serious diseases like cancers. In the present review, we discuss environmental and genetic factors that cause changes in microbiota composition, disposing of the host towards cancer, and the molecular mechanisms (such as β-catenin signalling) and biochemical pathways (like the generation of oncogenic metabolites like N-nitrosamines and hydrogen sulphide) that the microbiota uses to initiate or accelerate cancers, with emphasis on gastrointestinal cancers. Moreover, we discuss how microbiota can adversely influence the success of colorectal-cancer chemotherapy, and its role in tumour metastasis. We also attempted to resolve conflicting results obtained for the butyrate effect on tumour suppression in the colon, often referred to as the ‘butyrate paradox’. In addition, we suggest the development of microbiota-based biomarkers for early cancer diagnosis, and a few target molecules of which the inhibition can increase the overall chances of cancer cure.

## 1. Introduction

There are about 100 trillion symbiotic microbial cells in the human body [1]. This gene pool of the host microbiota is referred to as its microbiome and is composed of the bacteriome (bacterial gene pool), the virome (viral gene pool), and the mycobiome (fungal gene pool) [2]. Our microbiota is primarily composed of bacterial cells (~99%). The gut alone has as many bacteria as the total number of cells in the human body [3,4]. The mechanism by which the host bacteriome triggers, promotes, or alleviates cancer is subtler than that of viruses, and only partially understood. An insignificant small number of bacteria may act as a principal carcinogen; one such example is *Helicobacter pylori* [5]. 

Humans encounter various microbes at various stages of their life. A neonate first receives its microbiota from the mother. In this regard, the mode of delivery of the infant (vaginal versus C-section) also plays a role in shaping its microbiota, as the mode of delivery determines the first exposure and composition of a neonate’s microbiota [6,7,8,9,10]. The microbiota and the neonate’s immune system, through intricate interactions, configure each other further in life [11,12,13]. This is a delicate process leading to homeostasis.

## 2. Microbiota and Host Immunity

Epithelial barriers and certain regulatory proteins like immunoglobulin A (IgA) and regenerating islet-derived 3 gammas (RegIIIγ) play a crucial role in maintaining a healthy symbiotic relationship between host and its microbiota. RegIIIγ is an antibacterial lectin that forms a physical barrier between the gut epithelium and the microbiota, and prevents immune reactions and inflammation. Microbiota-derived products were reported to contribute to steady-state haematopoiesis and myelopoiesis [14,15,16]. More factors governing the relationship between microbiota and host immunity are discussed below. 

## 3. Microbiota Eubiosis: Characteristics and Implications

Microbiota–immune homeostasis is a state called microbiota eubiosis, and the emergent microbiota is termed as eubiotic microbiota, which confers important benefits related to physical and mental health, and the development of an individual. The dynamics of microbiota during life mainly depend upon the host’s genetics [17,18], environment, lifestyle [19], and dietary habits [20]. Changes in any of these factors can profoundly alter the gut microbiota. A negative alteration in the gut microbiota that increases the host’s propensity towards diseases is termed as microbiota dysbiosis [21]. This dysbiosed microbiota breaks down the delicate homeostasis that existed between eubiotic commensals and immune system. These events can align the cells and tissue towards inflammation, resulting in long-term consequences, such as inflammatory bowel disease (IBD) or accelerated tumourigenesis in cancer patients.

**Figure 1:**
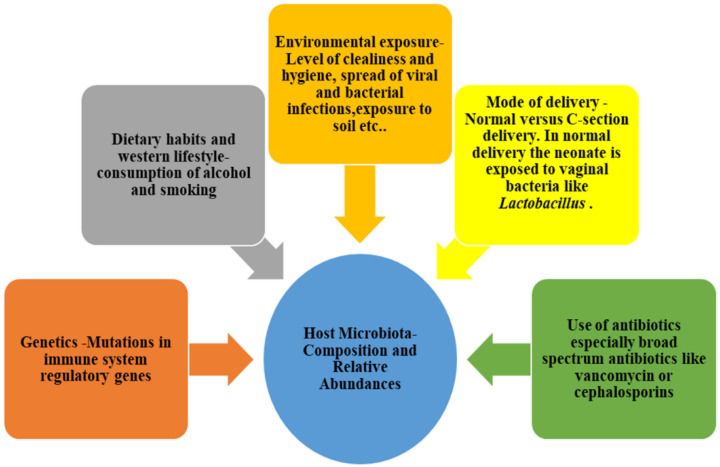
Factors contributing to shaping and changing gut microbiota with time.

Major factors that lead to microbiota dysbiosis include the early exposure to antibiotics in life, or the too-frequent use of broad-spectrum antibiotics [22,23,24], unhealthy habits like alcoholism and smoking [25], excessive consumption of a modern high-fat and high-protein and low-fibre diet (referred to as the westernised diet), and genetic mutations in regulatory genes such as sirtuins [26], nucleotide-binding oligomerisation-domain (NLRP) genes [27], mucin 2 [28,29,30], and lipocalin 2 (Lcn2) [31] (Figure 1). Most of these regulatory genes control barriers between microbiota and host tissue, thus preventing inflammation. These regulatory genes also play a key role in shaping the composition of the gut microbiota. The gain of function mutation in NLRP3 was reported to positively contribute to microbiota composition, leading to a microbiota that induces anti-inflammatory state and prevents colorectal cancer (CRC). Lcn2 deficiency increases iron-bound siderophores, such as enterobactin, in the intestines that cause an overgrowth of *Alistipes spp*. that assimilate siderophore-bound iron for their growth. *Alistipes spp.,* which are prominent carcinogenic bacteria [32], were proven to induce right-sided tumours in the intestine [31,33,34].

Microbial dysbiosis in the context to cancer refers to changes in total microbial load, in the relative abundance of various microbial taxa, and in the operational taxonomic units in a particular location with time [35]. All these observations led to the proposition of various microbiota-related biomarkers for the early detection of cancers [36].

Complexity in identifying and rectifying a dysbiotic microbiota, with a tendency to drive the process of tumourigenesis, springs from observations that, in a tumour microenvironment, the microbiota changes with time as the tumour progresses through various stages. It then becomes difficult to ascertain whether the bacterial population in the current microbiota is responsible for the initiation or the potentiation of cancer, or is associated with the site just because it finds the tumour niche better for its survival. For example, a hypoxic tumour niche may select for an anaerobic bacterial population. In the case of nonintestinal cancers, drawing associations between microbiota and cancer becomes even more complex [37], as the location of the culprit microbiota and cancer is usually different (dysbiosed gut microbiota can promote tumourigenesis in the liver or breast [38,39], as discussed in Table 1).

## 4. Dysbiosed Microbiota as Tumour Promoter

**Figure 2:**
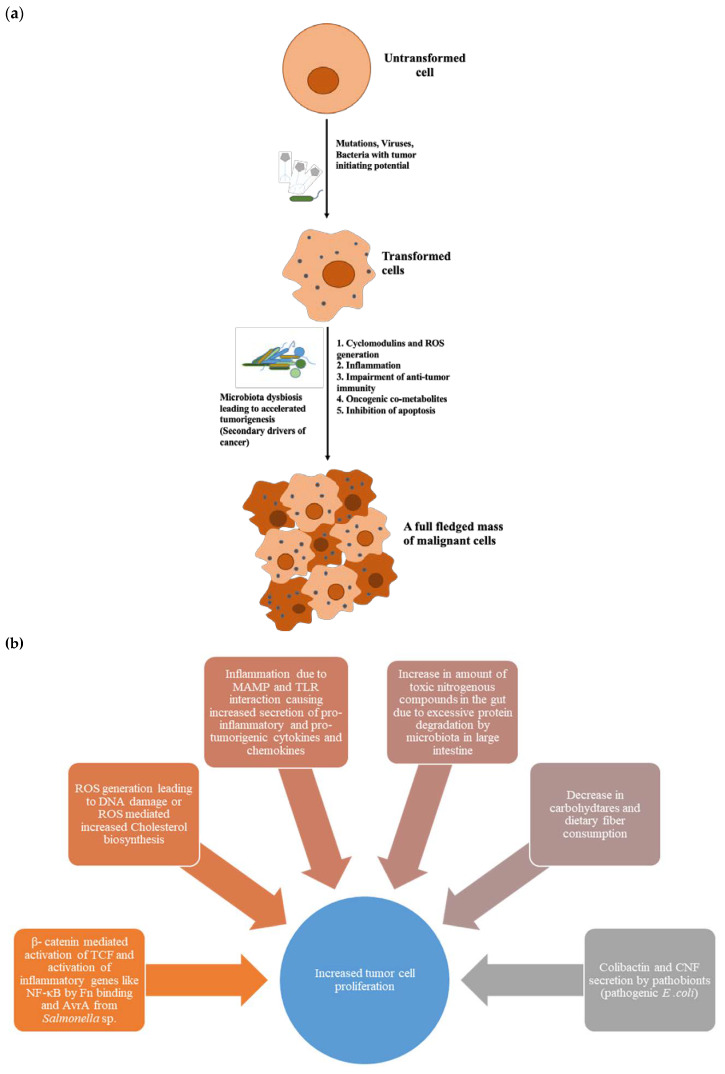
Overview of microbiota-driven cancer. (**a**) Microbiota acts as secondary driver in tumourigenesis, (**b**) factors increasing the tumor cell proliferation.

Both in vivo and in vitro studies, as well as xenograft murine models, have brought out some common mechanisms that a microbiota that has gone astray employs in the pathogenicity of different kinds of cancer in its host. These mechanisms are summarised in Figure 2 and Table 1. A bacterial clade that finds a neoplastic environment suitable for its survival can use more than one mechanism at a time to speed up the process of tumourigenesis. Some bacterial clades are even involved in cancer metastasis and recurrence after chemotherapy. The major tools and mechanisms that the dysbiosed microbiota uses are as follows.

### 4.1. Cyclomodulins

Oncogenic bacterial clades can disrupt cell polarity and cytoskeletal structure, and also tip the balance between cell proliferation and death. They can achieve this through cyclomodulins, which are genotoxins or effectors that can modulate the eukaryotic-cell cycle [64].

A common example is the cyclomodulins-mediated activation of the Wnt–β-catenin pathway [65]. Activated β-catenin forms a complex with the Tcf family of transcription factors that can upregulate the expression of oncogenes like c-myc [66]. Cyclomodulins that promote cell proliferation generally interact with the Rho family of GTPases such as Gln61, which can then disrupt cytoskeletal structures and relay mitogenic signals inside the cells [67]. Cytotoxic necrotizing factor (CNF) released by *E. coli* [47,68] and *Yersinia pseudotuberculosis* [69], dermonecrotic toxin by *Bordetella sp*. [70], *Pastreulla multocida* toxin (PMT) [71], and cytotoxin-associated gene A (Cag A) from *H. pylori* [72] are cyclomodulins that increase cell proliferation: PMT inhibits apoptosis in tumour cells, Cag A can also cause epithelial-barrier disruption, and the *Bacteroides fragilis* toxin indirectly damages DNA by generating reactive oxygen species (ROS) [44]. 

### 4.2. Microbial-Associated Molecular Patterns (MAMPs) and Inflammation

MAMPs are microbe-derived chemical motifs, such as surface molecules and endotoxins. A very common example of a MAMP is the lipopolysaccharide (LPS) layer of Gram-negative bacteria. Pattern-recognition receptors (PRRs), especially toll-like receptors (TLRs) like TLR-4 and TLR-5 of innate immunity, recognise MAMPs and mount a suitable immune response against a microbe. Members of a dysbiosed microbiota in the tumour microenvironment activate certain TLRs by their MAMPs. The activated TLRs can elicit an inflammatory response in the region of the tumour via interleukins such as IL-23, IL-17, IL-18, and IL-6 [73]. These inflammatory interleukins further promote the growth of tumour cells, inhibit apoptosis, and harm neighbouring normal cells through transcription factor NF-κB. TLR4 does not only exert a proinflammatory response; rather, in an underacetylated condition, LPS molecules from predominant gut species like *Bacteroides* on interaction with TLR4 mostly exert an immunoinhibitory effect [74]. For better understanding of the relationship of microbiota and host immunity, please see the recent review by Giuffre et al. and references therein [74].

### 4.3. Oncogenic Microbial Metabolites

Oral and gut microbiota play a significant role in host metabolism. A dysbiosed microbiota can disrupt natural host metabolism and physiology, and lead to the production of metabolites that drive tumourigenesis and impair antitumour immunity. An example of this is the conversion of primary into secondary bile acids such as deoxycholic acids by dysbiosed Gram-positive bacterial strains decreasing natural-killer T (NKT) cell populations and accelerating hepatocellular-carcinoma (HCC) [74,75] development [76].

## 5. Gut Microbiota and Gastrointestinal Cancers: Potential Molecular Mechanisms

Ample studies have established the important role of gut-microbiota dysbiosis in the development of gastrointestinal-tract cancers. Metagenomics studies revealed that many oral bacteria or oral bacterial biofilms become associated with gastrointestinal malignancies in the initial stages, and assist in accelerating tumourigenesis [77]. *Fusobacterium nucleatum* (Fn) is a Gram-negative, anaerobic, oral commensal that makes use of the haematogenous route to reach tumour cells in the gut from the oral cavity. Other common oral bacterial clades found in intestinal malignancies include *Prevotella* and *Parvimonas.*

Inflammatory conditions like IBD, Crohn’s disease [78], colitis, and cancerous genetic mutations like the adenomatous polyposis coli gene (APC^min/+^), mismatch repair gene (Msh2^–/–^), and B-Raf proto-oncogene (BRAF) mutations are often associated with pro-oncogenic gut microbiota that may include bacteria like enterotoxigenic *Bacteroides fragilis* (ETBF) [45,46] and Fn [79,80,81].

### 5.1. Cancer Initiation and Progression in Stomach

Unlike the intestines, our stomach is considered sterile due to its highly acidic environment. Bacteria in the stomach cannot remain there and are in a passage from mouth to intestine along with food [82]. However, the Gram-negative *H. pylori* penetrates the gastric mucosa and establishes itself there. It then increases the pH of its environment by secreting urease that leads to decreased acid secretion and achlorhydria, changing the gastric microbiota. *H. pylori* also induces a strong inflammatory response and releases CagA (120–145 kDa protein) and peptidoglycan into the cellular environment. CagA on phosphorylation in the gastric mucosa disrupts the cytoskeletal structure and cell–cell junctions. Peptidoglycan activates the phosphatidylinositol 3-kinase (PI3K-Akt) signalling pathway, resulting in the inhibition of cell apoptosis and enhanced cellular migration. *H. pylori* thus transforms normal gut epithelial cells into malignant cells [83,84,85,86].

According to a study on the Taiwanese population [40], the abundance of *H. pylori* in the transformed cells is diminished and replaced by other bacteria like Fn, *Streptococcus, Prevotella, Veillonella, Burkholderia, Nitrospirae, Escherichia,* and *Shigella.* which could further drive tumourigenesis [60,61]. Another computational study found that the most common bacterial DNA that is integrated into transformed cells of gastric adenocarcinomas belongs to *Pseudonomas* [62]. It can thus be hypothesised that prolonged *H. pylori* infection could increase the chances of colonisation of other tumour-accelerating bacteria due to achlorhydria.

### 5.2. Cancer Initiation and Progression in Intestine

Intestines harbour many symbiotic bacteria like *Lactobacillus* and *Lachnospiraceae*. These bacteria help in proper digestion, producing anti-inflammatory effects and preventing the colonisation of the intestines by oral bacterial clades. However, studies indicate that the absence of these beneficial bacteria, and microbiota dysbiosis in the intestine can lead to an imbalance of the immune system, diabetes [63], obesity [87], IBD, and intestinal cancers. The enumeration of intestinal microbiota dysbiosis, especially that of the large intestine, can provide us with better insights into the development, metastasis, and recurrence of colorectal cancer (CRC). The severity of CRC in different patients, which is dependent on the location of the tumour in the intestines [88] or the resistance intensity to chemotherapy, can be better determined by understanding host–microbiota interactions in CRC [36,89]. The impact of different dietary and lifestyle habits on the chances of CRC development in the entire population is also dependent on intestinal microbiota than any other factor [90,91].

Some of the most important bacteria that play a key role in the pathogenesis of CRC are Fn, *Bacteroides fragilis,* and *E. coli*. A dysbiosed microbiota can accelerate tumourigenesis in the colon by converting neoplastic polyps into adenomas and adenocarcinomas. This increased fecundity of tumour polyps is a result of the microbiota’s ability to upregulate oncogenes and elicit inflammation [92,93,94]. Tumour cells in CRC are uniquely overexpressed on their surface receptors, such as E-cadherin and galactose/N-acetylgalactosamine (Gal–GalNAc) lectin. These receptors have an affinity for two virulent adhesion proteins, FadA and Fap2, present on the surface of Fn.

**Figure 3:**
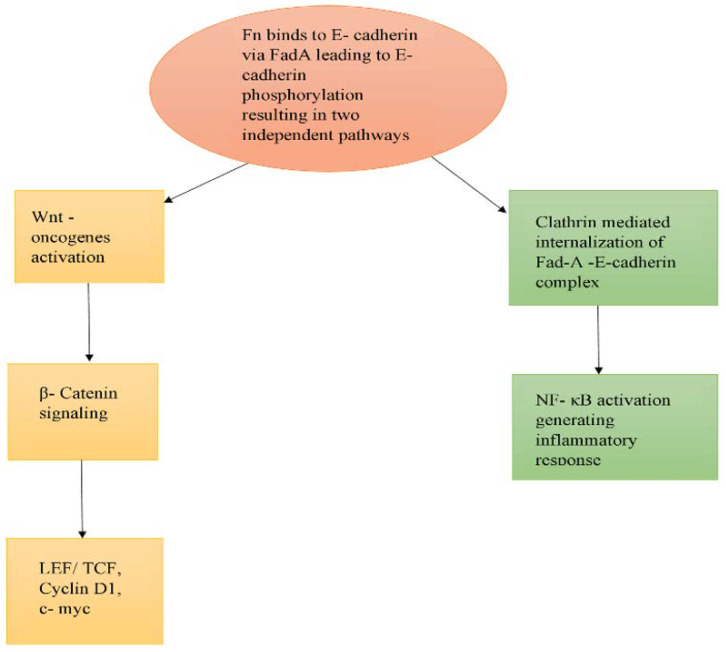
Pathway depicting FadA-mediated tumourigenesis by *Fusobacterium nucleatum* (Fn).

Fn binds to E-cadherin receptors via FadA and could activate Wnt oncogenes and inflammatory genes [42]. E-cadherin–FadA interaction leads to the immediate phosphorylation of E-cadherin, and then to its internalisation into the cytoplasm and the activation of β-catenin signalling. β-catenin signalling then activates the LEF/TCF, oncogenes Myc and cyclin D1, and NF-κB, the prime driver of inflammation. Although β-catenin signalling is required for the activation of oncogenes and NF-κB, E-cadherin–FadA internalisation is mandatory for the activation of inflammatory genes, but not oncogenes, as shown in Figure 3. This internalisation of E-cadherin and FadA is carried by clathrin, and inhibition of clathrin leads to failure of activation of inflammatory genes. 

Abed et al. showed a different way by which Fn adheres to tumour cells in the intestine, i.e., through polysaccharide Gal–Gal–NAc that is overexpressed on those tumour cells [43]. Lectin Fap2 on the surface of Fn helps in this binding. This interaction also triggers the acceleration of tumourigenesis, but the molecular basis of this interaction still needs to be understood. Fn is an oral bacterium that employs the haematogenous route to reach neoplastic cells in the intestine, where the required haemagglutination for transport is also mediated by Fap2 [95]. 

A study also reported that Fn interacts with TLR 4, and activates NF-κB and miRNA-21, leading to increased cell proliferation in CRC cells [96]. Fn probably finds a tumour niche suitable for its better growth because of the hypoxic conditions existing inside the tumour mass. Adherent molecules FadA and Fap2 provide Fn with an evolutionary advantage to be selected for this conducive tumour niche. This is evident from the fact that other oral anaerobes, such as *P. gingivalis,* associated with oral carcinoma are unable to adhere and persist in the tumour niche of CRC even though the tumour microenvironment is also suitable for the growth of *P. gingivalis* [43].

Besides Fn, *E. coli* is a common commensal bacterium of the human intestines that prevents the colonisation of other pathogenic bacteria in the intestine. However, certain strains of *E. coli* are themselves pathogenic and referred to as pathobionts that are commonly associated with tumour cells in CRC; recent studies have confirmed their role not only in CRC acceleration, but also in its establishment [97]. The general mechanisms by which pathogenic *E. coli* drives cancers are via toxic cyclomodulins like CNF, as discussed above, and by disrupting the DNA repair mechanism of the host cells. The most profound of these toxins is colibactin encoded by the polyketide synthase (pks) locus of the pks^+^ *E. coli* [48,98]. The pks^+^ *E. coli* induces cell senescence via colibactin, but, at a low multiplicity of infection (MOI), this effect can promote tumourigenesis. At an MOI of 20, the pks^+^ *E. coli* via colibactin activates c-Myc that, in turn, activates the promoter regions of miRNA 20a-5p. These miRNAs can cause the SUMOylation of *p53* genes (inhibiting p53 activity) and downregulate the expression of sentrin-specific protease 1 (*SENP1)*. All this leads to the senescence of cells that then release growth factors, the most important of which is hepatocyte growth factor (HGF), into the tumour microenvironment, leading to the accelerated growth of neighbouring tumour cells [99]. However, at a higher MOI of 100, colibactin-induced cellular senescence can completely inhibit tumour-cell proliferation and reduce their numbers due to cell senescence. 

#### 5.2.1. Microbiota-Driven Suppression of Antitumour Immunity

The gut microbiome is not only capable of triggering and accelerating tumourigenesis, but it can also help a tumour successfully evade an immune response against it. NK cells with other tumour-infiltrating lymphocytes (TILs) are mainly responsible for destroying tumour cells by identifying specific ligands through their activating receptors. On the other hand, the activity of these immune cells is kept in check by various inhibitory receptors on their surface. An example of these receptors is the T-cell immunoglobulin and ITIM domain (TIGIT) receptor that is expressed on a majority of TILs, including NK cells and T lymphocytes. Fn, so frequently associated with adenomas and adenocarcinomas, can dampen this antitumour response of TILs by binding its Fap2 protein to the TIGIT receptor present on their surface [100]. Fap2 thus plays the dual role of tumour-cell adhesion molecule and TIL inhibitor via haemagglutination. Some microbial-derived products can be immunosuppressive, and some can provoke an immune response against cancer, as seen in the case of Coley’s toxins [101].

#### 5.2.2. Microbiota Role in CRC Metastasis and Recurrence 

Loss of contact inhibition and disruption of cell–cell adhesion molecules are important prerequisites for the epithelial-to-mesenchymal transition (EMT) and metastasis of tumour cells. In altered-microbiota-driven CRC, Fn that is highly prevalent in CRC tissue binds to the E-cadherin expressed on adenomas and adenocarcinomas, leading to its internalisation into the cytoplasm, and the activation of the β-catenin complex and inflammatory genes. The E-cadherin/β-catenin complex is involved in cell adhesion, morphogenesis, polarity, migration, and development. The activation of the β-catenin complex leads to EMT and the metastasis of various solid tumours. Therefore, Fn-mediated activation of the β-catenin complex can incline CRC tumour cells towards metastasis. 

Fn is one of the primary reasons for CRC metastasis in many patients and it carries itself along with other oral anaerobes to distant sites to find a new niche. Other oral commensals like *Bacteroides, Salmonella,* and *Prevotella* that are present, along with Fn, in CRC tissue are incapable of tumour-cell metastasis by themselves, and are therefore dependent on Fn.

In addition to initiating metastasis, a high persistence of Fn was found in malignant cells of recurrent CRC patients. Drugs like oxaliplatin and 5-fluorouracil (5-FU) combined are one of the most common chemotherapeutic regimens used for the treatment of CRC. However, a lot of patients show recurrence after treatment with this regimen due to drug resistance, resulting in a five-year survival rate of less than 10% in such patients. Fn, even at low MOI, is one of the major factors responsible for conferring chemotherapeutic resistance against oxaliplatin and 5-FU by modulating the autophagy pathway in tumour cells [102]. Fn, via TLR4 and its signalling adapter molecule MyD88, can suppress the expression of miR18* and miR4802 in the cell. The suppression of these miRNAs causes upregulation in the expression of two proteins, Unc-51-like autophagy-activating kinase (ULK1) and autophagy-related 7(ATG7). The activation of the autophagy pathway thus helps tumour cells to be preserved through all the toxicity and stress inflicted upon them by the chemotherapeutic regimen. Therefore, adverse alterations in the gut microbiota can not only trigger and accelerate CRC, but also cause its metastasis and recurrence. One of the ways of reducing tumourigenic microbial load is by treatment with antibiotics. Metronidazole administration was shown to decrease Fn abundance, causing a reduction in cell proliferation and tumour load [103].

#### 5.2.3. Dietary Habits, Host–Microbiome Cometabolism, and the Butyrate Paradox 

Our normal gut microbiota depends upon the fermentation of the indigestible dietary-fibre component of our diet for its energy requirements. The symbiotic intestinal microbiota ferments dietary fibres into short-chain fatty acids (SCFAs) such as butyrate, acetate, and propionate, of which butyrate is the most important [91]. These SCFAs have significant anti-inflammatory and immunomodulatory functions, and thus protect an individual from CRC [104,105,106].

Acetate induces IgA activation by interacting with G-protein-coupled (GPR43) receptors present on adipose tissue in the intestine [107]. IgA plays an integral role in the modulation of gut microbiota and maintaining microbiota eubiosis by binding to pathogenic bacteria (expressing certain IgA-targeted epitopes) and suppressing their growth [108,109,110,111]. Another study reported that IgA promotes the symbiosis of beneficial gut bacteria [112]. A decrease in dietary-fibre intake coupled with the increased consumption of a westernised diet (low-dietary-fibre, and high-protein and high saturated-fat) [113] leads to a drastic decline in healthy and probiotic gut microbiota, and paves the way for other opportunistic carcinogenic pathogens [104] such as invasive pks^+^ *E. coli* [114]. In a study, less dietary-fibre consumption was associated with the increased expression of miRNA 17–92a clusters on colonic-cancer-cell lines. MiRNAs 17–92a are involved in the upregulation of tumour-inducing genes [115,116,117].

**Figure 4:**
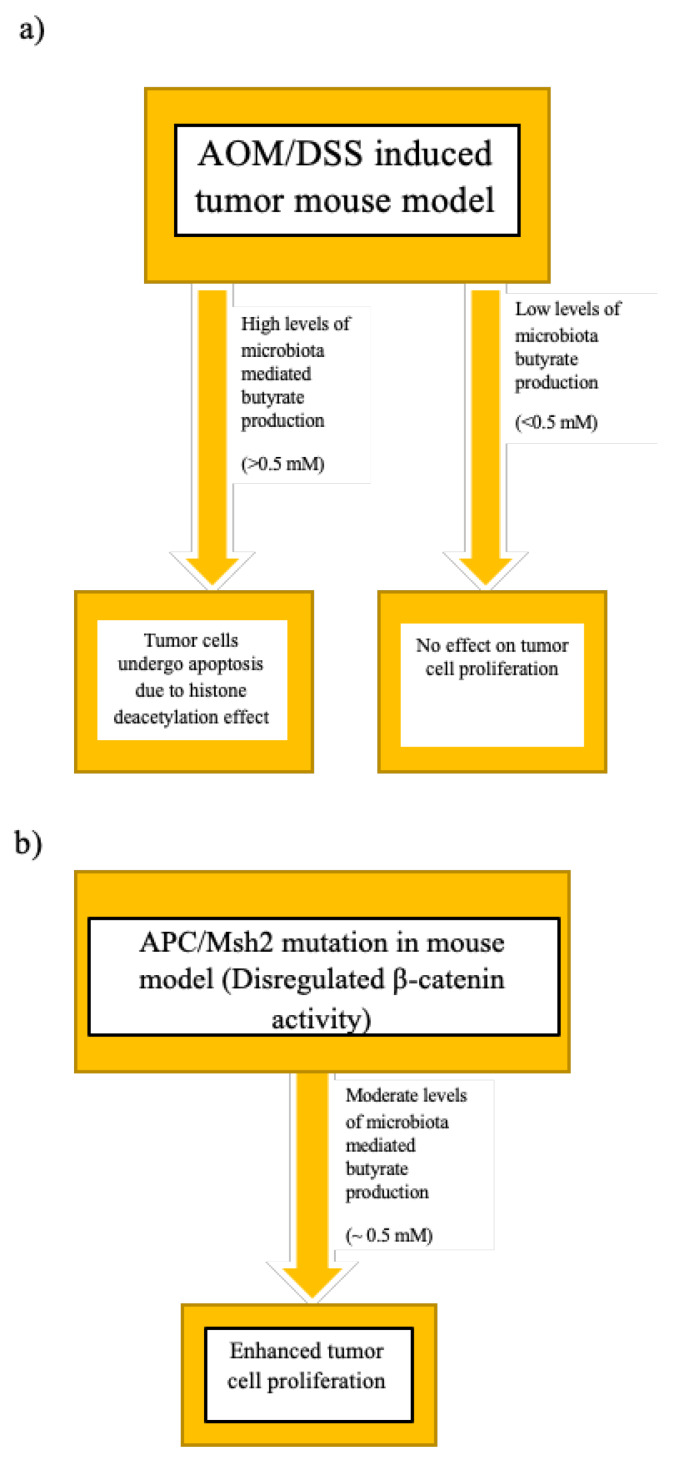
Butyrate paradox. (**a**) Effect of different levels of butyrate in Azoxymethane/Dextran sodium sulphate (AOM/DSS) treated mouse model. DSS is a colon epithelium disrupting agent that can induce colitis and AOM is a genotoxin. Combined AOM and DSS treatment in mice creates a model similar to that of human CRC to study the effects of inflammation and underlying genetic mutations in colon epithelium cells on microbiota dysbiosis and tumourigenesis [127]; (**b**) effect of moderate levels of butyrate on adenomatous polyposis coli (APC)/ MutS protein homolog 2 (Msh2) mouse model.

Butyrate inhibits colonocyte proliferation, and promotes apoptosis by the inhibition of histone deacteylase [118,119] and the canonical Wnt signalling pathway [120]. Butyrate maintains an anti-inflammatory state in the colon (via anti-inflammatory cytokines FOXP3 and IL-10) by interacting with the GPR 41 and GPR 43 receptors [121,122,123]. Butyrate also regulates CD4^+^ and CD8^+^ T_reg_ cells [124]. CD8^+^ T cells further mediate the apoptosis of tumours cells by employing IFN-γ and granzyme-B production [124]. However, not all studies agreed on the tumour-suppressing effect of butyrate, and some reported the opposite, i.e., butyrate could further promote tumourigenesis in neoplastic cells. For instance, in an APC and MutS protein homolog 2 (Msh2, a DNA mismatch repair protein) gene-mutation-driven model of CRC, butyrate accelerated the proliferation of neoplastic cells [125]; this was termed as the butyrate paradox. In the APC/Msh2 mouse model, there was high dysregulation of Wnt–β-catenin activity [125]. The intensity of Wnt–β-catenin activity is directly related to the amount of butyric acid produced by the colonic microbiota. Very high Wnt–β-catenin activity leads to cell apoptosis. However, a moderate amount of butyrate maintains the Wnt–β-catenin activity required for cell proliferation [126]. It can thus be hypothesised that, in the model of Belcheva et al., moderate levels of butyrate production coupled with dysregulated β-catenin lead to enhanced tumour-cell proliferation (Figure 4). 

Hydrogen sulphide (H_2_S) produced in the gut can cause colonic inflammation and cancers [128]. H_2_S in the colon can induce tumourigenesis by causing DNA damage and the inhibition of butyrogenesis. The major source of H_2_S in the gut is sulphur-reducing δ-proteobacteria that include *Desulfovibrio* and *Desulfobulbus*. Bacteria capable of the desulfhydration of cysteine and methionine, such as *H. pylori*, *E. coli,* and *Enterococci*, and assimilatory sulphite-reducing γ-proteobacteria such as *Klebsiella*, Firmicutes, and Bacteriodites are also some of other sources of H_2_S in the gut. A high abundance of these H_2_S-producing bacteria in the gut can dispose of the host towards H_2_S-mediated colonic inflammation and cancer.

An excessive amount of proteins in the diet can lead to malabsorption of proteins in the small intestine, which can lead to an excess of protein seepage into the large intestine, where the gut microbiota of the large intestine converts these proteins into metabolites such as N-nitrosamines, ammonia, and hydrogen sulphide, which are all potentially carcinogenic [129]. *Lactobacillus spp.* can detoxify ammonia generated in the large intestine [130], but dysbiosis leading to a decreased abundance of *Lactobacillus* can increase CRC risk due to ammonia. 

## 6. Conclusions

All recent studies now convincingly put *Fusobacterium nucleatum* on equal stature to the already infamous *Helicobacter pylori* for its role in cancer. Future research must now discover more such culprits from our oral and gut microbiota. Moreover, we need to focus on mitigating the impact of microbiota on cancer causation, acceleration, metastasis, and failure to chemotherapy through the development of targeted drug-delivery options. Some of the targets for such an approach can be β-catenin molecules in tumour cells, IL-6, and related TLRs in areas of severe inflammation and microbiota dysbiosis in the body; molecules like E-cadherin and Gal–GalNac that are overexpressed on tumour cells and act as binding sites for bacteria or their effector molecules; and miRNAs involved in the autophagy of tumour cells and their increased proliferation. Another area of extensive research is to develop microbiota-based biomarkers for the early diagnosis of cancers. This is based on the fact that early stages of cancer development and progression are accompanied by specific changes in microbiota populations (as certain operational taxonomic units increase or diminish as they elicit an altered immune response, increased oxidative stress, and changes in cometabolism). Quantitative assessment of specific microbial products (colibactin, AvrA, etc.) from cells/tissue or oncogenic cometabolites from faecal samples can act as biomarkers for cancer diagnostics. These biomarkers should not only be a determinant of current microbiota status, but should also be able to indicate relative changes in microbiota populations over time. RT–PCR-based analysis of microbiota populations, DNA biosensors strips, ELISA-based or faecal immunochemical test-based technologies can be developed to monitor changes in biomarkers in specific cells/tissue, and blood or stool samples. Continuous monitoring of changes in microbiota profiles may thus help in the identification of dysplasia. A more complete and holistic approach towards treating diseases as severe as cancer must include host–microbiota interactions as important screening and cure factors.

## Figures and Tables

**Table 1:** Different pathogenic bacteria/bacterial clades, their possible mode of action, and cancer types with these bacteria. List is not exhaustive, but brings out some common modes of action by different bacterial clades in pathogenesis of different cancers.

Bacteria/Clade	Mode of Action	Cancer
*Helicobacter pylori*	Disruption of stomach and colonic epithelial integrity creates a niche in stomach suitable for further pathogenic bacterial invasion [40].	Stomach and colorectal.
*Fusobacterium nucleatum*	Suspension of disintegration of β-catenin signalling, increased expression of TLR4 activation of p21- activated kinase and cyclin D1 [41], increased inflammatory gene expression, and suppression of antitumour NKT cells via effector molecules FadA and Fap2 [42,43].	Stomach, colorectal, oral, and lung.
*Bacteroides fragilis*	Reactive-oxygen-species (ROS) generation leading to DNA damage, colon-epithelial-barrier disruption, and depletion of mucous membrane, causing increased inflammation [44,45,46].	Stomach, colorectal, and lung.
Pathogenic *Escherichia coli*	Toxin colibactin indirectly induces release of growth factors in tumour microenvironment; cytotoxic necrotizing factor (CNF)-mediated disruption of host cell DNA repair mechanism [47,48].	Stomach and lung.
*Salmonella sp*.	Stabilises and prevents degradation of β-catenin by deubiquitinase activity of its AvrA protein [49,50,51].	Stomach, colorectal, gall-bladder, and lung.
*Peptostreptococcus anaerobius*	Increases expression of SREB2 gene via ROS, causing increased cholesterol biosynthesis in colon [52].	Colorectal.
*Citrobacter rodentium*	Loss of cell polarity, depletion of epithelial barrier, and increased inflammation [53].	Colorectal.
*Mycobacterium tuberculosis, Streptococcus viridans, Haemophilus influenza, Streptococcus pnuemoniae, Staphyloccocus*	Involved in various chronic inflammatory lung disorders like asthma, cystic fibrosis, and chronic obstructive pulmonary disease; potential for accelerating tumourigenesis via inflammatory cytokines like tumour necrosis factor [54,55,56].	Lung.
*Porphyromonas gingivalis* and *Aggregatibacter actinomycetemcomitans*	Reach pancreas from oral cavity through blood circulation and act as secondary drivers of cancer; impair host innate immunity, leading to increased colonisation by other bacteria, leading to chronic inflammation of pancreas causing accelerated tumourigenesis [57,58,59].	Pancreatic.
Proteobacteria, Betaproteobacteria, Firmicutes, Alcaligenaceae, Burkholderiales	Alter metabolism and oestrogen recycling, and exert pressure on immune system [38].	Breast.
*P. gingivalis* and *Tannerella forsythia*	Cause overexpression of inflammatory cytokines; gingipain K produced by *P. gingivalis* paralyses immune cells, and induce indirect overexpression of glucose-transporter (GLUT-1 and GLUT-4) genes that help in faster tumour-cell proliferation [60,61,62,63].	Oesophageal.

## References

[1] Sender R., Fuchs S., Milo R. (2016). Revised Estimates for the Number of Human and Bacteria Cells in the Body. PLoS Biol..

[2] Limon J.J., Skalski J.H., Underhill D.M. (2017). Commensal Fungi in Health and Disease. Cell Host Microbe.

[3] Backhed F. (2005). Host–Bacterial Mutualism in the Human Intestine. Science.

[4] Gill S.R., Pop M., DeBoy R.T., Eckburg P.B., Turnbaugh P.J., Samuel B.S., Gordon J.I., Relman D.A., Fraser-Liggett C.M., Nelson K.E. (2006). Metagenomic Analysis of the Human Distal Gut Microbiome. Science.

[5] Plummer M., de Martel C., Vignat J., Ferlay J., Bray F., Franceschi S. (2016). Global burden of cancers attributable to infections in 2012: A synthetic analysis. The Lancet Global Health.

[6] Bennet R., Nord C.E. (1987). Development of the Faecal Anaerobic Microflora After Caesarean Section and Treatment with Antibiotics in Newborn Infants. Infection.

[7] Dominguez-Bello M.G., Costello E.K., Contreras M., Magris M., Hidalgo G., Fierer N., Knight R. (2010). Delivery mode shapes the acquisition and structure of the initial microbiota across multiple body habitats in newborns. PNAS.

[8] Dominguez-Bello M.G., De Jesus-Laboy K.M., Shen N., Cox L.M., Amir A., Gonzalez A., Bokulich N.A., Song S.J., Hoashi M., Rivera-Vinas J.I. (2016). Partial restoration of the microbiota of cesarean-born infants via vaginal microbial transfer. Nat. Med..

[9] Palmer C., Bik E.M., Digiulio D.B., Relman D.A., Brown P.O. (2007). Development of the Human Infant Intestinal Microbiota. PLoS Biol..

[10] Roswall J., Peng Y., Feng Q., Jia H., Kovatcheva-datchary P. (2015). Dynamics and Stabilization of the Human Gut Microbiome during the First Year of Life Resource. Cell Host Microbe.

[11] Francis S.S., Selvin S., Metayer C., Wallace A.D., Crouse V., Moore T.B., Wiemels J.L., Buffler P.A. (2014). Mode of delivery and risk of childhood leukemia. Cancer Epidemiol Biomarkers Prev..

[12] Gensollen T., Iyer S.S., Kasper D.L., Blumberg R.S. (2016). How colonization by microbiota in early life shapes the immune system. Science.

[13] Tamburini S., Shen N., Wu H.C., Clemente J.C. (2016). The microbiome in early life: Implications for health outcomes. Nature Publishing Group.

[14] Maslowski K.M., Vieira A.T., Ng A., Kranich J., Sierro F., Di Y., Schilter H.C., Rolph M.S., Mackay F., Artis D. (2009). Regulation of inflammatory responses by gut microbiota and chemoattractant receptor GPR43. Nature.

[15] Shi C., Jia T., Mendez-Ferrer S., Hohl T.M., Serbina N.V., Lipuma L., Leiner I., Li M.O., Frenette P.S., Pamer E.G. (2011). Bone Marrow Mesenchymal Stem and Progenitor Cells Induce Monocyte Emigration in Response to Circulating Toll-like Receptor Ligands. Immunity.

[16] Tada T., Yamamura S., Kuwano Y., Abo T. (1996). Level of Myelopoiesis in the Bone Marrow Is Influenced by Intestinal Flora. Cell Immunol..

[17] Gomez A., Espinoza J.L., Harkins D.M., Leong P., Saffery R., Bockmann M., Torralba M., Kuelbs C., Kodukula R., Inman J. (2017). Host Genetic Control of the Oral Microbiome in Health and Disease. Cell Host Microbe.

[18] Qin J., Li Y., Cai Z., Li S., Zhu J., Zhang F., Liang S., Zhang W., Guan Y., Shen D. (2012). A metagenome-wide association study of gut microbiota in type 2 diabetes. Nature.

[19] David L.A., Materna A.C., Friedman J., Campos-Baptista M.I., Blackburn M.C., Perrotta A., Erdman S.E., Alm E.J. (2014). Host lifestyle affects human microbiota on daily timescales. Genome Biol..

[20] Graf D., Di Cagno R., Fåk F., Flint H.J., Nyman M., Saarela M., Watzl B. (2015). Contribution of diet to the composition of the human gut microbiota. Microb Ecol..

[21] Sommer F., Anderson J.M., Bharti R., Raes J., Rosenstiel P. (2017). The resilience of the intestinal microbiota influences health and disease. Nat. Rev. Microbiol.

[22] Cao Y., Wu K., Mehta R., Drew D.A., Song M., Lochhead P., Nguyen L.H., Izard J., Fuchs C.S., Garrett W.S. (2017). Long-term use of antibiotics and risk of colorectal adenoma. Gut.

[23] Freifeld A.G., Bow E.J., Sepkowitz K.A., Boeckh M.J., Ito J.I., Mullen C.A., Raad I.I., Rolston K.V., Young J.-A.H., Wingard J.R. (2011). Clinical Practice Guideline for the Use of Antimicrobial Agents in Neutropenic Patients with Cancer: 2010 Update by the Infectious Diseases Society of America. Clin. Infect. Dis..

[24] Isaac S., Scher J.U., Djukovic A., Jiménez N., Littman D.R., Abramson S.B., Pamer E.G., Ubeda C. (2017). Short- and long-term effects of oral vancomycin on the human intestinal microbiota. J. Antimicrob. Chemother..

[25] Capurso G., Lahner E. (2017). The interaction between smoking, alcohol and the gut microbiome. Best Pr. Res. Clin. Gastroenterol..

[26] Zhang Y., Wang X., Zhou M., Kang C., Lang H., Chen M., Hui S., Wang B., Mi M. (2018). Crosstalk between gut microbiota and Sirtuin-3 in colonic inflammation and tumorigenesis. Exp. Mol. Med..

[27] Yao X., Zhang C., Xing Y., Xue G., Zhang Q., Pan F., Wu G., Hu Y., Guo Q., Lu A. (2017). Remodelling of the gut microbiota by hyperactive NLRP3 induces regulatory T cells to maintain homeostasis. Nat. Commun..

[28] Kumar M., Kissoon-singh V., Coria A.L., Moreau F., Chadee K. (2017). Probiotic mixture VSL # 3 reduces colonic inflammation and improves intestinal barrier function in Muc2 mucin-deficient mice. Am J Physiol Gastrointest Liver Physiol..

[29] Velcich A. (2002). Colorectal Cancer in Mice Genetically Deficient in the Mucin Muc2. Science.

[30] Wu M., Wu Y., Li J., Bao Y., Guo Y., Yang W. (2018). The Dynamic Changes of Gut Microbiota in Muc2 Deficient Mice. Int. J. Mol..

[31] Moschen A.R., Gerner R.R., Wang J., Klepsch V., Adolph T.E., Reider S.J., Hackl H., Pfister A., Schilling J., Moser P.L. (2016). Lipocalin 2 Protects from Inflammation and Tumorigenesis Associated with Gut Microbiota Alterations. Cell Host Microbe.

[32] Dai Z., Coker O.O., Nakatsu G., Wu W.K.K., Zhao L., Chen Z., Chan F.K.L., Kristiansen K., Sung J.J.Y., Wong S.H. (2018). Multi-cohort analysis of colorectal cancer metagenome identified altered bacteria across populations and universal bacterial markers. Microbiome.

[33] Ellermann M., Arthur J.C. (2017). Siderophore-mediated iron acquisition and modulation of host–bacterial interactions. Free Radic. Biol. Med..

[34] Feng Q., Liang S., Jia H., Stadlmayr A., Tang L., Lan Z., Zhang D., Xia H., Xu X., Jie Z. (2015). Gut microbiome development along the colorectal adenoma–carcinoma sequence. Nat. Commun.

[35] Flemer B., Warren R.D., Barrett M.P., Cisek K., Das A., Jeffery I.B., Hurley E., Riordain M.O., Shanahan F., Toole P.W.O. (2017). The oral microbiota in colorectal cancer is distinctive and predictive. Gut.

[36] Flemer B., Lynch D.B., Brown J.M.R., Jeffery I.B., Ryan F.J., Claesson M.J., O’Riordain M., Shanahan F., O’Toole P.W. (2017). Tumour-associated and non-tumour-associated microbiota in colorectal cancer. Gut.

[37] Jacqueline C., Brazier L., Faugère D., Renaud F., Roche B. (2017). Can intestinal microbiota be associated with non-intestinal cancers?. Sci. Rep..

[38] Goedert J.J., Hua X., Bielecka A., Okayasu I., Milne G.L., Jones G.S., Fujiwara M., Sinha R., Wan Y., Xu X. (2018). Postmenopausal breast cancer and oestrogen associations with the IgA-coated and IgA-noncoated faecal microbiota. Br. J. Cancer.

[39] Fernández M.F., Reina-Pérez I., Astorga J.M., Rodríguez-Carrillo A., Plaza-Díaz J., Fontana L. (2018). Breast Cancer and Its Relationship with the Microbiota. Int. J. Environ. Res. Public Health.

[40] Hsieh Y.Y., Tung S.Y., Pan H.Y., Yen C.W., Xu H.W., Lin Y.J., Deng Y.F., Hsu W.T., Wu C.S., Li C. (2018). Increased Abundance of Clostridium and Fusobacterium in Gastric Microbiota of Patients with Gastric Cancer in Taiwan. Sci. Rep..

[41] Wu Y., Wu J., Chen T., Li Q., Peng W., Li H., Tang X., Fu X. (2018). Fusobacterium nucleatum Potentiates Intestinal Tumorigenesis in Mice via a Toll-Like Receptor 4/p21-Activated Kinase 1 Cascade. Dig. Dis Sci.

[42] Rubinstein M.R., Wang X., Liu W., Hao Y., Cai G., Han Y.W. (2013). Fusobacterium nucleatum Promotes Colorectal Carcinogenesis by Modulating E-Cadherin/β-Catenin Signaling via its FadA Adhesin. Cell Host Microbe.

[43] Abed J., Emgård J.E.M., Zamir G., Faroja M., Almogy G., Grenov A., Sol A., Naor R., Pikarsky E., Atlan K.A. (2016). Fap2 Mediates Fusobacterium nucleatum Colorectal Adenocarcinoma Enrichment by Binding to Tumor-Expressed Gal-GalNAc. Cell Host Microbe.

[44] Goodwin A.C., Shields C.E.D., Wu S., Huso D.L., Wu X., Murray-Stewart T.R., Hacker-Prietz A., Rabizadeh S., Woster P.M., Sears C.L. (2011). Polyamine catabolism contributes to enterotoxigenic Bacteroides fragilis-induced colon tumorigenesis. PNAS.

[45] Sears C.L., Geis A.L., Housseau F. (2014). Bacteroides fragilis subverts mucosal biology: From symbiont to colon carcinogenesis. J. Clin. Invest..

[46] Wu S., Rhee K.-J., Albesiano E., Rabizadeh S., Wu X., Yen H.-R., Huso D.L., Brancati F.L., Wick E., McAllister F. (2009). A human colonic commensal promotes colon tumorigenesis via activation of T helper type 17 T cell responses. Nat. Med..

[47] Bonnet M., Buc E., Sauvanet P., Darcha C., Dubois D., Pereira B., Dechelotte P., Bonnet R., Pezet D., Darfeuille-Michaud A. (2014). Colonization of the Human Gut by E. coli and Colorectal Cancer Risk. Clin. Cancer Res..

[48] Cuevas-Ramos G., Petit C.R., Marcq I., Boury M., Oswald E., Nougayrede J.-P. (2010). Escherichia coli induces DNA damage in vivo and triggers genomic instability in mammalian cells. PNAS.

[49] Lu R., Wu S., Zhang Y., Xia Y., Liu X., Zheng Y., Chen H., Schaefer K.L., Zhou Z., Bissonnette M. (2014). Enteric bacterial protein AvrA promotes colonic tumorigenesis and activates colonic beta-catenin signaling pathway. Oncogenesis.

[50] Di Domenico E.G., Cavallo I., Pontone M., Toma L., Ensoli F. (2017). Biofilm Producing Salmonella Typhi: Chronic Colonization and Development of Gallbladder Cancer. Int. J. Mol. Sci..

[51] Koshiol J., Wozniak A., Cook P., Adaniel C., Acevedo J., Azócar L., Hsing A.W., Roa J.C., Pasetti M.F., Miquel J.F. (2016). *Salmonella enterica* serovar Typhi and gallbladder cancer: A case-control study and meta-analysis. Cancer Med..

[52] Tsoi H., Chu E.S.H., Zhang X., Sheng J., Nakatsu G., Ng S.C., Chan A.W.H., Chan F.K.L., Sung J.J.Y., Yu J. (2017). Peptostreptococcus anaerobius Induces Intracellular Cholesterol Biosynthesis in Colon Cells to Induce Proliferation and Causes Dysplasia in Mice. Gastroenterology.

[53] Newman J.V., Kosaka T., Sheppard B.J., Fox J.G., Schauer D.B. (2001). Bacterial Infection Promotes Colon Tumorigenesis in *Apc*
^Min/+^ Mice. J. Infect. Dis..

[54] Mao Q., Jiang F., Yin R., Wang J., Xia W., Dong G., Ma W., Yang Y., Xu L., Hu J. (2018). Interplay between the lung microbiome and lung cancer. Cancer Lett..

[55] Pilaniya V., Gera K., Kunal S., Shah A. (2016). Pulmonary tuberculosis masquerading as metastatic lung disease. Eur. Respir. Rev..

[56] Liang H.-Y., Li X.-L., Yu X.-S., Guan P., Yin Z.-H., He Q.-C., Zhou B.-S. (2009). Facts and fiction of the relationship between preexisting tuberculosis and lung cancer risk: A systematic review. Int. J. Cancer.

[57] Fan X., Alekseyenko A.V., Wu J., Peters B.A., Jacobs E.J., Gapstur S.M., Purdue M.P., Abnet C.C., Stolzenberg-Solomon R., Miller G. (2018). Human oral microbiome and prospective risk for pancreatic cancer: A population-based nested case-control study. Gut.

[58] Curtis M.A. (2014). Periodontal Microbiology—The Lid’s off the Box Again. J. Dent. Res..

[59] Jia G., Zhi A., Lai P.F.H., Wang G., Xia Y., Xiong Z., Zhang H., Che N., Ai L. (2018). The oral microbiota – a mechanistic role for systemic diseases. Br. Dent. J..

[60] Dicksved J., Lindberg M., Rosenquist M., Enroth H., Jansson J.K., Engstrand L. (2009). Molecular characterization of the stomach microbiota in patients with gastric cancer and in controls. J. Med. Microbiol..

[61] Wang L., Zhou J., Xin Y., Geng C., Tian Z., Yu X., Dong Q. (2016). Bacterial overgrowth and diversi fi cation of microbiota in gastric cancer. Eur. J. Gastroenterol. Hepatol..

[62] Robinson K.M., Crabtree J., Mattick J.S.A., Anderson K.E., Hotopp J.C.D. (2017). Distinguishing potential bacteria-tumor associations from contamination in a secondary data analysis of public cancer genome sequence data. Microbiome.

[63] Tremaroli V., Bäckhed F. (2012). Functional interactions between the gut microbiota and host metabolism. Nature.

[64] El-Aouar Filho R.A., Nicolas A., De Paula Castro T.L., Deplanche M., De Carvalho Azevedo V.A., Goossens P.L., Taieb F., Lina G., Le Loir Y., Berkova N. (2017). Heterogeneous Family of Cyclomodulins: Smart Weapons That Allow Bacteria to Hijack the Eukaryotic Cell Cycle and Promote Infections. Front. Cell. Infect. Microbiol..

[65] Man S.M., Zhu Q., Zhu L., Liu Z., Karki R., Malik A., Sharma D., Li L., Malireddi R.K.S., Gurung P. (2015). Critical Role for the DNA Sensor AIM2 in Stem Cell Proliferation and Cancer. Cell.

[66] Wu S., Morin P.J., Maouyo D., Sears C.L. (2003). Bacteroides fragilis enterotoxin induces c-Myc expression and cellular proliferation. Gastroenterology.

[67] Harmey D., Stenbeck G., Nobes C.D., Lax A.J., Grigoriadis A.E. (2004). Regulation of Osteoblast Differentiation by Pasteurella Multocida Toxin (PMT): A Role for Rho GTPase in Bone Formation. J. Bone Miner. Res..

[68] Buc E., Dubois D., Sauvanet P., Raisch J., Delmas J., Darfeuille-Michaud A., Pezet D., Bonnet R. (2013). High Prevalence of Mucosa-Associated, E. coli Producing Cyclomodulin and Genotoxin in Colon Cancer. PLoS ONE.

[69] Nougayrède J.-P., Taieb F., Rycke J.D., Oswald E. (2005). Cyclomodulins: Bacterial effectors that modulate the eukaryotic cell cycle. TIM.

[70] Horiguchi Y. (2001). Escherichia coli cytotoxic necrotizing factors and Bordetella dermonecrotic toxin: The dermonecrosis-inducing toxins activating Rho small GTPases. Toxicon.

[71] Lax A., Aktories K., Orth J.H.C., Adler B. (2012). The Pasteurella multocida Toxin: A New Paradigm for the Link Between Bacterial Infection and Cancer. Pasteurella multocida.

[72] De Luca A., Iaquinto G. (2004). Helicobacter pylori and gastric diseases: A dangerous association. Cancer Letters.

[73] Hu B., Elinav E., Huber S., Strowig T., Hao L., Hafemann A., Jin C., Wunderlich C., Wunderlich T., Eisenbarth S.C. (2013). Microbiota-induced activation of epithelial IL-6 signaling links inflammasome-driven inflammation with transmissible cancer. PNAS.

[74] Giuffrè M., Campigotto M., Campisciano G., Comar M., Crocè L.S. (2020). A Story of Liver and Gut Microbes: How Does the Intestinal Flora Affect Liver Disease?. AM. J. Physiol.-Gastrointest. Liver Physiol..

[75] Yu L.-X., Schwabe R.F. (2017). The gut microbiome and liver cancer: Mechanisms and clinical translation. Nat. Rev. Gastroenterol Hepatol..

[76] Ma C., Han M., Heinrich B., Fu Q., Zhang Q., Sandhu M., Agdashian D., Terabe M., Berzofsky J.A., Fako V. (2018). Gut microbiome – mediated bile acid metabolism regulates liver cancer via NKT cells. Science.

[77] Drewes J.L., White J.R., Dejea C.M., Fathi P., Iyadorai T., Vadivelu J., Roslani A.C., Wick E.C., Mongodin E.F., Loke M.F. (2017). High-resolution bacterial 16S rRNA gene profile meta-analysis and biofilm status reveal common colorectal cancer consortia. npj Biofilms Microbiomes.

[78] Pascal V., Pozuelo M., Borruel N., Casellas F., Campos D., Santiago A., Martinez X., Varela E., Sarrabayrouse G., Machiels K. (2017). A microbial signature for Crohn’s disease. Gut.

[79] Kostic A.D., Chun E., Robertson L., Glickman J.N., Gallini C.A., Michaud M., Clancy T.E., Chung D.C., Lochhead P., Hold G.L. (2013). Fusobacterium nucleatum Potentiates Intestinal Tumorigenesis and Modulates the Tumor-Immune Microenvironment. Cell Host Microbe.

[80] Mima K., Nishihara R., Qian Z.R., Cao Y., Sukawa Y., Nowak J.A., Yang J., Dou R., Masugi Y., Song M. (2016). *Fusobacterium nucleatum* in colorectal carcinoma tissue and patient prognosis. Gut.

[81] Tahara T., Yamamoto E., Suzuki H., Maruyama R., Chung W., Garriga J., Jelinek J., Yamano H.-o., Sugai T., An B. (2014). Fusobacterium in Colonic Flora and Molecular Features of Colorectal Carcinoma. Cancer Res..

[82] Ianiro G., Molina-Infante J., Gasbarrini A. (2015). Gastric Microbiota. Helicobacter.

[83] Tegtmeyer N., Wessler S., Backert S. (2011). Role of the cag -pathogenicity island encoded type IV secretion system in Helicobacter pylori pathogenesis. FEBS J..

[84] Tegtmeyer N., Backert S. (2011). Role of Abl and Src family kinases in actin-cytoskeletal rearrangements induced by the Helicobacter pylori CagA protein. Eur. J. Cell Biol..

[85] Wu J., Xu S., Zhu Y. (2013). Helicobacter pylori CagA: A Critical Destroyer of the Gastric Epithelial Barrier. Dig. Dis. Sci..

[86] Tohidpour A. (2016). CagA-mediated Pathogenesis of Helicobacter pylori. Microb. Pathog..

[87] Ley R.E., Turnbaugh P.J., Klein S., Gordon J.I. (2006). Human gut microbes associated with obesity. Nature.

[88] Aoyama T., Kashiwabara K., Oba K., Honda M., Sadahiro S., Hamada C., Maeda H., Mayanagi S., Kanda M., Sakamoto J. (2017). Clinical impact of tumor location on the colon cancer survival and recurrence: Analyses of pooled data from three large phase III randomised clinical trials. Cancer Med..

[89] Mima K., Cao Y., Chan A.T., Qian Z.R., Nowak J.A., Masugi Y., Shi Y., Song M., da Silva A., Gu M. (2016). Fusobacterium nucleatum in Colorectal Carcinoma Tissue According to Tumor Location. Clin. Transl. Gastroenterol..

[90] Arnold M., Sierra M.S., Laversanne M., Soerjomataram I., Jemal A., Bray F. (2017). Global patterns and trends in colorectal cancer incidence and mortality. Gut.

[91] O’Keefe S.J.D. (2016). Diet, microorganisms and their metabolites, and colon cancer. Nat. Rev. Gastroenterol. Hepatol..

[92] Arthur J.C., Perez-Chanona E., Mühlbauer M., Tomkovich S., Uronis J.M., Fan T.-J., Campbell B.J., Abujamel T., Dogan B., Rogers A.B. (2012). Intestinal Inflammation Targets Cancer-Inducing Activity of the Microbiota. Science.

[93] Garrett W.S. (2015). Cancer and the microbiota. Science.

[94] Schwabe R.F., Jobin C. (2013). The microbiome and cancer. Nat. Rev. Cancer.

[95] Coppenhagen-Glazer S., Sol A., Abed J., Naor R., Zhang X., Han Y.W., Bachrach G. (2015). Fap2 of Fusobacterium nucleatum Is a Galactose-Inhibitable Adhesin Involved in Coaggregation, Cell Adhesion, and Preterm Birth. Infect. Immun..

[96] Yang Y., Weng W., Peng J., Hong L., Yang L., Toiyama Y., Gao R., Liu M., Yin M., Pan C. (2017). Fusobacterium nucleatum Increases Proliferation of Colorectal Cancer Cells and Tumor Development in Mice by Activating Toll-Like Receptor 4 Signaling to Nuclear Factor−κB, and Up-regulating Expression of MicroRNA-21. Gastroenterology.

[97] Tomkovich S., Yang Y., Winglee K., Gauthier J., Mühlbauer M., Sun X., Mohamadzadeh M., Liu X., Martin P., Wang G.P. (2017). Locoregional Effects of Microbiota in a Preclinical Model of Colon Carcinogenesis. Cancer Res..

[98] Arthur J.C., Gharaibeh R.Z., Mühlbauer M., Perez-Chanona E., Uronis J.M., McCafferty J., Fodor A.A., Jobin C. (2014). Microbial genomic analysis reveals the essential role of inflammation in bacteria-induced colorectal cancer. Nat. Commun.

[99] Dalmasso G., Cougnoux A., Delmas J., Darfeuille-Michaud A., Bonnet R. (2014). The bacterial genotoxin colibactin promotes colon tumor growth by modifying the tumor microenvironment. Gut Microbes.

[100] Gur C., Ibrahim Y., Isaacson B., Yamin R., Abed J., Gamliel M., Enk J., Bar-On Y., Stanietsky-Kaynan N., Coppenhagen-Glazer S. (2015). Binding of the Fap2 Protein of Fusobacterium nucleatum to Human Inhibitory Receptor TIGIT Protects Tumors from Immune Cell Attack. Immunity.

[101] Nathenson M.J., Conley A.P., Sausville E. (2018). Immunotherapy: A New (and Old) Approach to Treatment of Soft Tissue and Bone Sarcomas. Oncologist.

[102] Yu T., Guo F., Yu Y., Sun T., Ma D., Han J., Qian Y., Kryczek I., Sun D., Nagarsheth N. (2017). Fusobacterium nucleatum Promotes Chemoresistance to Colorectal Cancer by Modulating Autophagy. Cell.

[103] Bullman S., Pedamallu C.S., Sicinska E., Clancy T.E., Zhang X., Cai D., Neuberg D., Huang K., Guevara F., Nelson T. (2017). Analysis of Fusobacterium persistence and antibiotic response in colorectal cancer. Science.

[104] Desai M.S., Seekatz A.M., Koropatkin N.M., Kamada N., Hickey C.A., Wolter M., Pudlo N.A., Kitamoto S., Terrapon N., Muller A. (2016). A Dietary Fiber-Deprived Gut Microbiota Degrades the Colonic Mucus Barrier and Enhances Pathogen Susceptibility. Cell.

[105] Sivaprakasam S., Gurav A., Paschall A.V., Coe G.L., Chaudhary K., Cai Y., Kolhe R., Martin P., Browning D., Huang L. (2016). An essential role of Ffar2 (Gpr43) in dietary fibre-mediated promotion of healthy composition of gut microbiota and suppression of intestinal carcinogenesis. Oncogenesis.

[106] Zitvogel L., Pietrocola F., Kroemer G. (2017). Nutrition, inflammation and cancer. Nat. Immunol..

[107] Wu W., Sun M., Chen F., Cao A.T., Liu H., Zhao Y., Huang X., Xiao Y., Yao S., Zhao Q. (2017). Microbiota metabolite short-chain fatty acid acetate promotes intestinal IgA response to microbiota which is mediated by GPR43. Mucosal Immunol..

[108] Bhattacharya N., Yuan R., Prestwood T.R., Penny H.L., DiMaio M.A., Reticker-Flynn N.E., Krois C.R., Kenkel J.A., Pham T.D., Carmi Y. (2016). Normalizing Microbiota-Induced Retinoic Acid Deficiency Stimulates Protective CD8 + T Cell-Mediated Immunity in Colorectal Cancer. Immunity.

[109] Lyu Y., Wu L., Wang F., Shen X., Lin D. (2018). Carotenoid supplementation and retinoic acid in immunoglobulin A regulation of the gut microbiota dysbiosis. Exp. Biol Med. (Maywood).

[110] Okai S., Usui F., Ohta M., Mori H., Kurokawa K., Matsumoto S., Kato T., Miyauchi E., Ohno H., Shinkura R. (2017). Intestinal IgA as a modulator of the gut microbiota. Gut Microbes.

[111] Peterson D.A., McNulty N.P., Guruge J.L., Gordon J.I. (2007). IgA Response to Symbiotic Bacteria as a Mediator of Gut Homeostasis. Cell Host Microbe.

[112] Nakajima A., Vogelzang A., Maruya M., Miyajima M., Murata M., Son A., Kuwahara T., Tsuruyama T., Yamada S., Matsuura M. (2018). IgA regulates the composition and metabolic function of gut microbiota by promoting symbiosis between bacteria. J. Exp. Med..

[113] Statovci D., Aguilera M., MacSharry J., Melgar S. (2017). The Impact of Western Diet and Nutrients on the Microbiota and Immune Response at Mucosal Interfaces. Front. Immunol..

[114] Agus A., Denizot J., Thévenot J., Martinez-Medina M., Massier S., Sauvanet P., Bernalier-Donadille A., Denis S., Hofman P., Bonnet R. (2016). Western diet induces a shift in microbiota composition enhancing susceptibility to Adherent-Invasive, *E. coli* infection and intestinal inflammation. Sci Rep..

[115] Fadaka A.O., Ojo B.A., Adewale O.B., Esho T., Pretorius A. (2018). Effect of dietary components on miRNA and colorectal carcinogenesis. Cancer Cell Int..

[116] Hu S., Dong T.S., Dalal S.R., Wu F., Bissonnette M., Kwon J.H., Chang E.B. (2011). The Microbe-Derived Short Chain Fatty Acid Butyrate Targets miRNA-Dependent p21 Gene Expression in Human Colon Cancer. PLoS ONE.

[117] Hu S., Liu L., Chang E.B., Wang J.-Y., Raufman J.-P. (2015). Butyrate inhibits pro-proliferative miR-92a by diminishing c-Myc-induced miR-17-92a cluster transcription in human colon cancer cells. Mol. Cancer.

[118] Donohoe D.R., Holley D., Collins L.B., Montgomery S.A., Whitmore A.C., Hillhouse A., Curry K.P., Renner S.W., Greenwalt A., Ryan E.P. (2014). A Gnotobiotic Mouse Model Demonstrates That Dietary Fiber Protects against Colorectal Tumorigenesis in a Microbiota- and Butyrate-Dependent Manner. Cancer Discov..

[119] Marks P.A., Richon V.M., Rifkind R.A. (2000). Histone Deacetylase Inhibitors: Inducers of Differentiation or Apoptosis of Transformed Cells. J. Natl. Cancer.

[120] Bordonaro M., Lazarova D.L., Sartorelli A.C. (2008). Butyrate and Wnt signaling: A possible solution to the puzzle of dietary fiber and colon cancer risk?. Cell Cycle.

[121] Hu Y., Le Leu R.K., Christophersen C.T., Somashekar R., Conlon M.A., Meng X.Q., Winter J.M., Woodman R.J., McKinnon R., Young G.P. (2016). Manipulation of the gut microbiota using resistant starch is associated with protection against colitis-associated colorectal cancer in rats. Carcin.

[122] Smith P.M., Howitt M.R., Panikov N., Michaud M., Gallini C.A., Bohlooly Y.M., Glickman J.N., Garrett W.S. (2013). The Microbial Metabolites, Short-Chain Fatty Acids, Regulate Colonic Treg Cell Homeostasis. Science.

[123] Tang Y., Chen Y., Jiang H., Robbins G.T., Nie D. (2011). G-protein-coupled receptor for short-chain fatty acids suppresses colon cancer. Int. J. Cancer.

[124] Luu M., Weigand K., Wedi F., Breidenbend C., Leister H., Pautz S., Adhikary T., Visekruna A. (2018). Regulation of the effector function of CD8+ T cells by gut microbiota-derived metabolite butyrate. Sci Rep..

[125] Belcheva A., Irrazabal T., Robertson S.J., Streutker C., Maughan H., Rubino S., Moriyama E.H., Copeland J.K., Surendra A., Kumar S. (2014). Gut Microbial Metabolism Drives Transformation of Msh2-Deficient Colon Epithelial Cells. Cell.

[126] Lazarova D.L., Bordonaro M., Carbone R., Sartorelli A.C. (2004). Linear relationship between Wnt activity levels and apoptosis in colorectal carcinoma cells exposed to butyrate. Int. J. Cancer.

[127] Baxter N.T., Zackular J.P., Chen G.Y., Schloss P.D. (2014). Structure of the gut microbiome following colonization with human feces determines colonic tumor burden. Microbiome.

[128] Linden D.R. (2013). Hydrogen Sulfide Signaling in the Gastrointestinal Tract. Antioxidants Redox Signal..

[129] Windey K., De Preter V., Verbeke K. (2012). Relevance of protein fermentation to gut health. Mol. Nutr. Food Res..

[130] Liu J., Lkhagva E., Chung H.-J., Kim H.-J., Hong S.-T. (2018). The Pharmabiotic Approach to Treat Hyperammonemia. Nutrients.

